# Involvement of *EGFR, ERK-1,2* and *AKT-1,2* Activity on Human Glioma Cell Growth

**DOI:** 10.31557/APJCP.2020.21.12.3469

**Published:** 2020-12

**Authors:** Amir Allahverdi, Ehsan Arefian, Masoud Soleimani, Jafar Ai, Aliakbar Yousefi-Ahmadipour, Abouzar Babaei, Md Shahidul Islam, Somayeh Ebrahimi-Barough

**Affiliations:** 1 *Department of Tissue Engineering and Applied Cell Sciences, School of Advanced Technologies in Medicine, Tehran University of Medical Sciences, Tehran, Iran. *; 2 *Stem Cell Technology Research Center, Tehran, Iran. *; 3 *Department of Microbiology, School of Biology, College of Science, University of Tehran, Tehran, Iran. *; 4 *Department of Hematology, Faculty of Medical Science, Tarbiat Modares University, Tehran, Iran. *; 5 *Department of Laboratory Sciences, School of Allied Medical Sciences, Rafsanjan University of Medical Sciences, Rafsanjan, Iran. *; 6 *Department of Virology, Faculty of Medical Science, Tarbiat Modares University, Tehran, Iran. *

**Keywords:** Glioblastoma, cell cycle, miR-4731, miRNAs

## Abstract

GBM (Glioblastoma multiforme) is the most prevalent and lethal primary brain tumor. Gene therapy is one of the promising approaches and involves the delivery of genetic therapeutic molecules for specific antitumour response/activity. miRNAs can regulate the cell biology functions including replication, cell growth, and apoptosis by regulating gene expression. In this study, we found that down-regulation of *miR-4731* expression occurred in GBM cells. We further determined that miR-4731 behaved as a tumor suppressor by inhibiting GBM cell proliferation. We further investigated the molecular mechanisms of *miR-4731* and *EGFR, ERK-1,2* and* AKT-1,2* in GBM cell lines U87 and U251. The *in vitro* ectopic expression of *miR-4731* affected cell proliferation, migration, and invasion of U87 and U251 cells. Luciferase reporter assays validated that miR-4731 targeted the 3′-untranslated region (3′-UTR) of EGFR. In conclusions, we identified that *miR-4731* plays a tumor suppressor role in GBM cell proliferation and migration by targeting *EGFR* expression, and *miR-4731* may act as a novel biomarker for early diagnosis or therapeutic target of GBM.

## Introduction

GBM (Glioblastoma multiforme) is the most prevalent and lethal primary malignant brain tumor (Ohgaki and Kleihues, 2013). The standard treatment method involves maximal resection of the tumor followed by radiation and chemotherapy (Wesseling and Capper, 2018). Gene therapy is one of the promising approaches and involves the delivery of genetic therapeutic molecules for specific antitumour response/activity(Manikandan et al., 2019). MicroRNA (miRNA) is a kind of non-coding RNA with the length of 19-23 nucleotides, which binds to the 3’ UTR of mRNA of the target genes to regulate the expression per se/their gene expression. miRNAs can interfere in transcription process of RNA and inhibit the expression of target genes (Valinezhad Orang et al., 2014). Many miRNAs have been known to perform specific functions in the regulation of tumor progression and multiple drug resistance (Shirjang et al., 2019). The 256 miRNAs were found to be increased and 95 miRNAs were reported as downregulated in GBM (Johnson et al., 2005). miR-4731 has been demonstrated to be a novel miRNA, which has been characterized as a tumor suppressor in human melanoma (Stark et al., 2016). However, the specific roles and regulatory mechanisms of miR-4731 in the progression of GBM have not been explored yet. Additionally, *EGFR* expression was identified to be highly expressed in a variety of human cancers, such as GBM (Huang et al., 2015). However, it is not clear whether miR-4731 can regulate the expression of *EGFR* in GBM.

In this study, we investigated the regulation of* EGFR,* *ERK-1,2* and *AKT* expression by miR-4731 and their effects on proliferation, cell cycle transition and invasion in glioblastoma cell lines U-251 and U-87. Our results may provide data for supporting miR-4731 as novel therapeutic tools for glioblastoma multiforme.

## Materials and Methods

*Bioinformatics analysis*

The KEGG database (https://www.genome.jp, RRID:SCR-012773) was obtained to accurately identify genes involved in the EGFR pathway. The target genes of miR-4731 were selected based on target scan algorithms. Expression Atlas database (https://www.ebi.ac.uk/gxa/home) was studied to determine the expression levels of *EGFR, ERK* and *AKT* genes in U-251MG and U-87MG cell lines.

*Cell culture and virus production*

Glioma cell line U-251MG was gifted from Stem Cell Technology Research Center (Tehran, Iran) and glioma cell line U-87MG (IBRC C10982) was purchased from Iranian Biological Resource Center (IBRC) (Tehran, Iran). Human embryonic kidney 293 T (IBRC C109683) was purchased from the Iranian Biological Resource Center (IBRC) (Tehran, Iran). All cells were routinely cultured in Dulbecco’s modified Eagle’s medium (DMEM, Bio Idea, Tehran, Iran) supplemented with 100 U/ml penicillin and 100 μg/ml streptomycin and 10% fetal bovine serum (FBS; Gibco). All cells were incubated at 37°C in a humidified atmosphere containing 5% CO_2_. For Virus production, HEK293 T cells were seeded in T-25 flask at 60–70% confluency. Virus packaging was performed using PEI transfection reagent (Polysciences, USA) according to the manufacturer’s protocol. hsa-miR-4731 recombinant vector and scramble vector co-transfected separately with psPAX and pMD2 vectors (psPAX and pMD2 virus packaging helper vectors were purchased from Stem Cell Technology Research Center (Tehran, Iran)) to HEK293 T cells and the culture media containing recombinant viral vectors was collected for 48, 72 and 96 hours. For in vitro functional assays, glioblastoma cells were seeded in T25 flask at 60–80% confluency. Transduction was performed by using Hexadimethrine bromide (commercial brand name Polybrene) as a transduction enhancer.

*Vectors and microRNA*

The *miR-4731* gene was amplified by PCR using specific primers (forward: 

5′- GCTCTAGACCAAGATTTCACCAGCACCAAG -3′ and reverse: 

5′CGGGATCCCATAGGACAGGCTCAATAGAGT TG -3′). 

This fragment was cloned into the pCDH-CMVMCS- EF1-cGFP-T2A-puro vector (System Biosciences, USA). The 3′UTR sequence of *EGFR* gene was amplified by PCR from genomic DNA using specific primers (forward: 5′- CCGCTCGAG CCA CGG AGG ATA GTA TGA GC-3′; and reverse: 5′- ATAAGAATGCGGCCGC CTTTCTTAACAATGCTGTAGGG -3′). These fragments were cloned into the psiCHECK-2 dual-luciferase reporter plasmid (Promega, Madison, WI, USA) downstream of the renilla luciferase gene.

*Reverse transcription-quantitative polymerase chain reaction (RT-qPCR)*

After 72 hours, total RNA from transduced glioma cells (U251 and U87MG) was extracted using GeneAll (Hybrid-R™) kit. The quality and concentration of total RNA were evaluated by spectrophotometry. To quantify *EGFR, ERK-1/2* and *miR-4731* expression, M-MLV reverse transcriptse (Thermofisher) was used for synthesis of single-stranded cDNA, and RT-qPCR was conducted using SYBR Green. *miR-4731* expression was calculated relative to *SNORD47* gene, and *EGFR, ERK-1/2* and *AKT* expressions were calculated relative to* β2M* gene. The primers in this study used for cDNA synthesis and real-time PCR were as follows in Table 2. Relative expression was evaluated by the 2^−ΔΔCt^ method.

*Luciferase reporter assay*

For the luciferase reporter technique, miR-4731 or scramble vector was transfected into hek-293T cells, along with EGFR-3′-UTR using PEI transfection reagent (Polysciences, USA) according to the manufacturer’s protocol. Luciferase activity was evaluated at 48 h post-transfection by a Dual-Luciferase Reporter assay system (Promega), considering to the manufacturer’s instructions. Firefly luciferase activity was obtained for normalization.

*Flow cytometry analysis*

Cell cycle was analyzed 72 hours after transduction. Transduced cells were collected by trypsinization, washed in cold PBS and fixed with paraformaldehyde. Then permeabilization of cells was performed with cold 70% ethanol, stained cells with dye including PI, PBS, Triton X100 and RNase A. After incubation at 37°C in the dark for 30 min, we assesed the samples on the flow cytometer BD FACS Calibur (BD Biosciences, San Jose, CA, USA).

*Colony forming Assay*

Briefly, U-87MG and U-251MG cells were seeded in 6 cm dishes at a concentration of 1,000 cells/well until colony formation. After 15 days, the supernatants were removed; then, colonies were carefully washed with PBS, fixed with cold methanol for 20 min, and stained with crystal violet 0.1% in PBS at room temperature for 10 min and air-dried. Images were captured and the total number of colonies/well was counted.

*Western blot analysis*

Cells transduced with LV-miR-4731 were collected, washed twice with cold phosphate-buffered saline (PBS) and resuspended in the RIPA lysis buffer with protease inhibitors (Roche Diagnostics GmbH) phosphatase inhibitors (Merck KGaA, Darmstadt, Germany). The total proteins was quantified using a BCA Protein assay kit (Pierce Biotechnology, Rockford, IL, USA). The same values of each protein sample were electrophoersed by 12 % SDS-polyacrylamide gel electrophoresis (SDS-PAGE) and transferred to polyvinylidine diflouride membranes (PVDF) (EMD Millipore, Billerica, MA, USA) and blocked with 5% fat-free milk. The membranes were then incubated overnight at 4°C with primary antibodies: Rabbit polyclonal Anti-ERK1,2 (EPR17526), Rabbit Anti-AKT1 + AKT2 + AKT3 antibody (ab126811) and Rabbit anti-human monoclonal Actin antibody (ab8227; 1:1000 dilution; Abcam; Cambridge; MA; UK). After washing three times with Trisbuffered saline with 0.5% Tween-20 (TBST; Beyotime Institute of Biotechnology, Haimen, China), the membranes were probed with Goat Anti-Rabbit IgG H&L (HRP) (ab6721; 1:5,000 dilution; Abcam; Cambridge; MA; UK) at room temperature for 1 h. The protein blots were visualized using the ECL Protein Detection kit (Pierce Biotechnology). The signal was detected using an enhanced chemiluminescence western blotting detection system.

*Statistical analysis*

The data in this study are presented as mean ± SD. Data were compared using Student’s t-test with GraphPad Prism 5.0 software and real-time PCR data was analyzed statistically by 2^−ΔΔCt^ method. Differences were assessed significant at P≤0.05.

**Figure 1:**
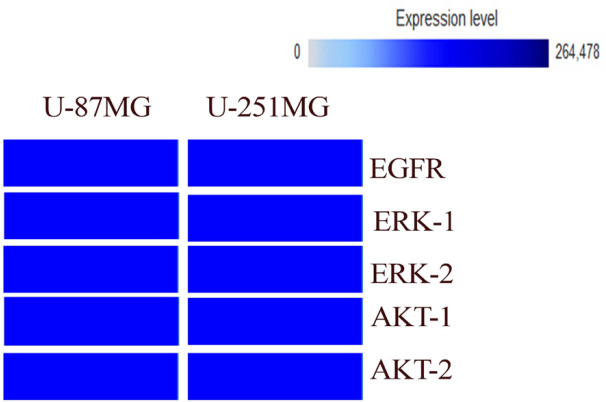
Comparison of the* EGFR, ERK-1,2* and *AKT-1,2* Expression in Tumoral (U87 MG and U251MG) and Non-Tumoral Samples. These data drived from the exprresion atlas database

**Table 1:** Primers Used for cDNA Synthesis and Real-Time PCR for *EGFR, AKT,1,2* and *ERK-1,2* Genes and hsa-miR-4731-5P

EGFR	Forward	CGTCCGCAAGTGTAAGAAG
	Reverse	AGGAGTCACCCCTAAATGC
AKT1	Forward	TGGCACCTTCATTGGCTAC
	Reverse	GTCTGGATGGCGGTTGTC
AKT2	Forward	ATTGCCAAGGATGAAGTCG
	Reverse	CTCAAGAGCCGAGACAATC
ERk1	Forward	GGTGGAGATGGTGAAGGG
	Reverse	CGCAGCAGGATCTGGATC
Β2M	Forward	ATGCCTGCCGTGTGAAC
	Reverse	ATCTTCAAACCTCCATGATG
Snord 47	Forward	GAGCAGGGTCCGAGGT
	RT primer	GTCGTATGCAGAGCAGGGTCCGAGGTATTCGCACTGCATACGACAACCTC
Common Reverse primer	Reverse	GAGCAGGGTCCGAGGT
hsa- miR-4731-5p	RT primer	GTCGTATGCAGAGCAGGGTCCGAGGTATTCGCACTGCATACGACCACACT
	Forward	TGCTGGGGGACACAT

**Figure 2:**
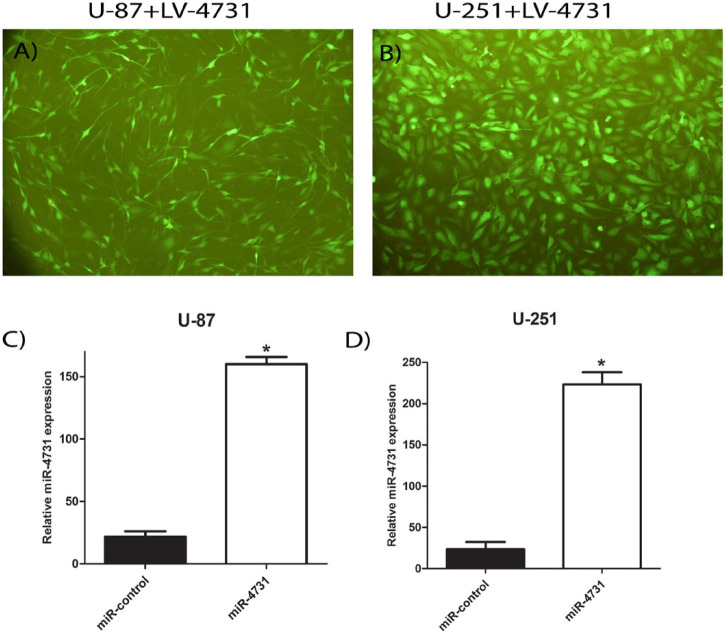
Establishment of mir-4731-Overexpressing Glioma Cell Lines. (A,B) GFP expression was observed under a fluorescence microscope after transduction. (A) U-87MG cells infected with lentiviral vectors expressing LV-miR-4731. (b) U-251MG cells infected with lentiviral vectors expressing LV-miR-4731. miR-4731 expression was analyzed by RT-PCR after infection in the U-87MG cells (C) and U-251MG (D). *P < 0.05 compared with control

**Figure 3:**
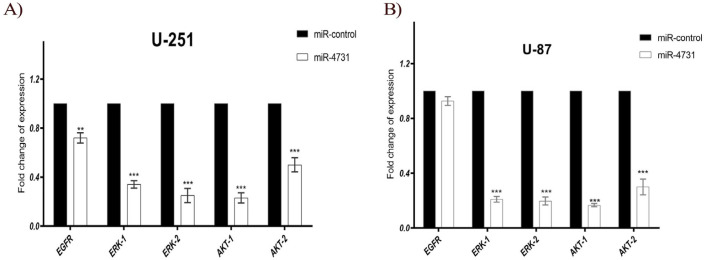
Real-Time PCR Analysis of Genes in *U-87MG* and *U-251MG. EGFR, ERK-1,2 *and *AKT-1,2* mRNA levels in U-87MG and U-251MG cells treated either with a miR-control or a miR-4731 after 72 h of transduction: ***P < 0.0001 Comparing with the control

**Figure 4:**
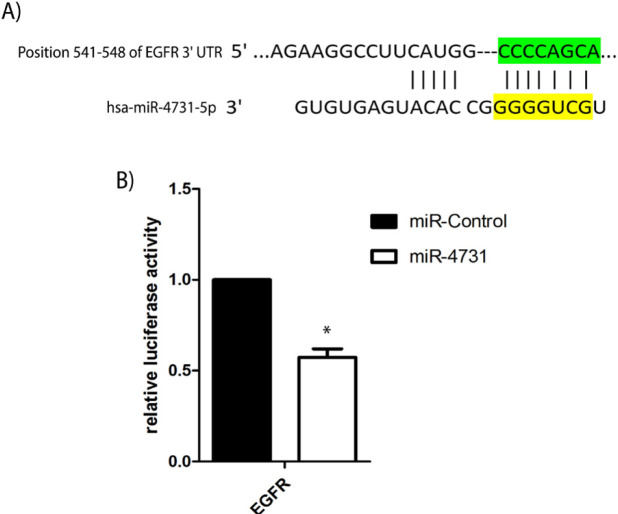
Luciferase Reporter Assay for the Interaction between miR-4731 and EGFR. (A) Predicted binding sites of hsa-miR-4731 in the 3'UTR of the EGFR mRNA. (B) Activity of the luciferase gene linked to the coding region of the EGFR mRNA

**Figure 5:**
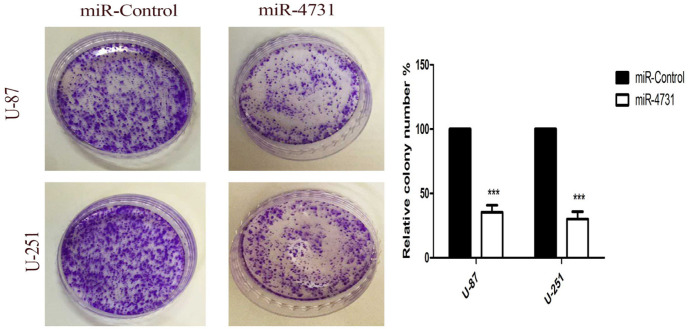
Colony Forming Ability of Glioma Cells Cultured after Infection with Lv-miR-4731 or LV-miR-control, Determined by a Colony Formation Assay. Overexpression of miR-4731 significantly decreases the proliferation ability of glioma cells. Plate colony formation assays on colony formation ability of control and miR-4731 expressing U-251MG and U-87MG cell. Graphical representation of colony counts in wells performed for each colony assay. Statistical analysis of the colony formation assays. ***P<0.0001 vs. control

**Figure 6:**
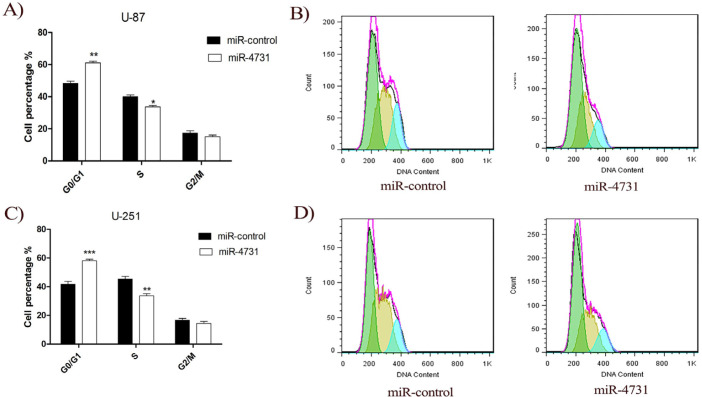
Cell Cycle Assay. MiR‐4731 induced the cell cycle arrest of human glioblastoma cell lines in vitro. The cell cycle analyzed with flow cytometry and PI staining, 72 hours after transduction. Overexpression of miR‐4731 significantly increased cell cycle arrest at G0/G1 phase in U-251MG and U-87MG. Data are exhibited as mean ± SD of results from three independent investigations (*P < 0.05, **P<0.001,***P<0.0001 ) vs. control

**Figure 7:**
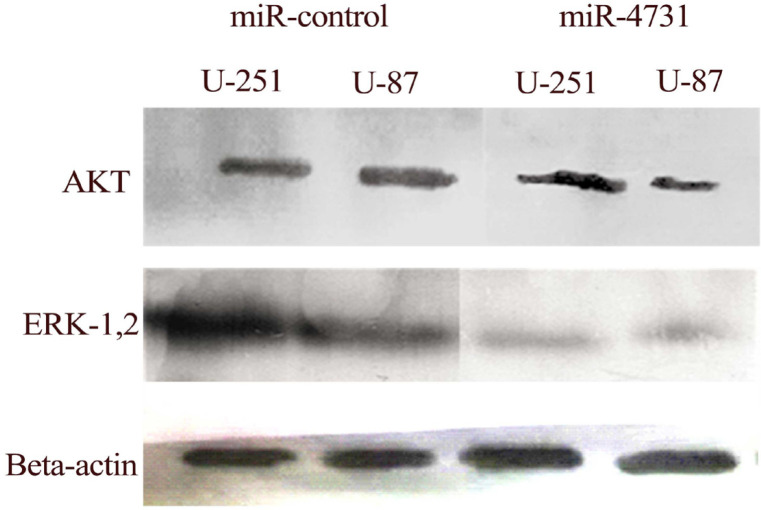
Downregulation of AKT and ERK-1,2 in U-251MG and U-87MG Cell Line. Western blot analysis was confirmed a decrease in AKT and ERK-1,2 protein expression in glioma cell line transduced with LV-miR-4731 compared with control cells

## Results

*miRNA and gene selection*

*EGFR, ERK-1,2* and *AKT*, were selected as target genes according to the KEGG database. hsa-miR-4731 that is targeted these genes was selected based on TargetScan algorithms. Moreover, based on Expression Atlas database, expression of *EGFR, ERK-1,2* and *AKT-1,2* in U-251 and U-87 cell lines were medium. In order to compare the expression level of the genes in the expression Atlas database, cerebral cortex were selected as non-tumoral tissue.The results of these evaluation indicated that the expression level of thess genes naturally in tumoral cells (U251MG and U87MG) is higher than non-tumoral cells (Figure 1).

*Induction of miR-4731 expression in glioblastoma cells*

After transfection of HEK293 T, LV-miR-4731 was collected and used for transduction of U-87MG (Figure2A) and U-251MG (Figure 2B) cell lines of glioblastoma. Then the expression of *miR-4731, EGFR, ERK-1,2* and *AKT-1,2* in two cell lines of glioblastoma (U-87MG and U-251MG) were evaluated by real-time PCR and compared with the glioblastoma cells transduced with the scramble vector as the control. The results showed the expression of *miR-4731 *in two cell lines and not in scramble group(Figure 2C, D).

*Expression of EGFR,ERK-1,2 and AKT-1,2 in glioblastoma cell lines*

we assessed expression of *EGFR, ERK-1,2* and *AKT-1,2* in U-251MG and U-87MG cells after 72 hours transduction with Lv-miR4731. In the independent experiments we observed marked reductions of EGFR, ERK-1,2 and AKT-1,2 transcript in glioma cells in response to miR-4731 infection (Figure 3). Of note, the EGFR mRNA levels were not decreased by miR-4731 over expression in *U-87MG*.

*miR-4731 directly targets and inhibits EGFR*

After the downregulation of *EGFR* expression as a result of the overexpression of *miR-4731* based on real-time PCR and the prediction of both of the genes targeting by miR-4731, luciferase reporter assay was performed to validate the binding miR-4731 and 3′-UTR of the *EGFR* gene. These findings indicated that overexpression of *miR-4731* can reduce the expression of the* EGFR* to ~66% (p < 0.0001). Therefore, it is suggested that miR-4731 directly targets and inhibits *EGFR* expression. (Figure 4).

*miR-4731 regulates proliferation of glioblastoma cells*

To investigate the effects of miR4731 on the proliferation of glioblastoma cells, by using colony formation assays, we observed that transduced U-251MG and U-87MG cells with miR-4731 dramatically decreased the growth rate of both types of glioblastoma cells as compared with that of the non-tranrasduced cells. miR-4731 decresed the growth rate of both types of glioblastoma cells as compared with that of the non-tranrasduced cells (Figure 4A and B). This suggests that up-regulation of miR-4731, which results in *EGFR, ERK-1,2* and *AKT-1,2* suppresion, decrease the proliferation of glioblastoma cells (Figure 5).

*Overexpression of miR-4731 induces cell cyle arrest in glioblastoma cells*

To investigate the effect of miR-4731 on the proliferation of glioblastoma cells, we transduced the glioblastoma cells with *LV-miR-4731* to increase its expression in these cells, then we analyzed the cell cycle by flow cytometry method. The miR-4731 resulted in significant increase in number of the cells in G0/G1 phase and reduces of cells in S phase (Figure 6). Our data indicate that miR-4731 induce G0/G1 cell cycle arrest in GBM cells.

*Downregulation of ERK-1,2 and AKT protein in glioblastoma*

The results of Western blot showed that the ERK-1,2 and AKT protein were down-regulated in U-87 MG and U-251MG cells transduced with LV-miR-4731 in comparison of miR-control groups. Our findings revealed that, ERK-1,2 and AKT protein expression decreased in the U-87 MG and U-251MG cells transduced with LV-miR-4731 compared with the control group (Figure 7).

## Discussion

Despite significant advances made over last years on molecular mechanisms invasion in glioblastoma, the exact reasons are still unknown and need more research. The treatment options for patients diagnosed with GBM are limited and the current median survival is 12-14 months following diagnosis (Kanu et al., 2009). Previous studies have shown that GBM frequently deliver mutations that activate EGFR and launch downstream signaling pathways, including the ATK and ERK molecuules (Mazzoleni et al., 2010). The EGFR signaling is activated in 60% of glioblastoma tumors and so, being a ‘critical molecule’ for glioblastoma (Maire and Ligon, 2014). The extracellular signal-regulated kinases (ERKs) is a signal transducer growth factor to the nucleus and involved in a wide range of biological responses, including cell proliferation, differentiation and motility. ERK1/2 is aberrantly expressed and activated in glioblastoma multiforme (Sun and Nan, 2017). AKT is a serine/threonine kinase in and large scale genomic analysis of GBM, has been demonstrated that this molecule is mutated in the majority of GBMs and inhibition of AKT an attractive target for GBM therapy (A McDowell et al., 2011).

The miRNAs can bind with 3 -’UTR region of target gene and suppress the of transcription of target gene. In cancers, the *miRNAs *expression patterns have been changed and alter the proliferative signaling, cell growth, invasion, and angiogenesis. EGFR pathway can also be regulated by many miRNAs. For example, miR-7 is down-regulated in human glioblastoma and directly targets *EGFR* expression. In addition, *miR-7* suppresses *Akt* and *ERK* pathway activation independent of its *EGFR* inhibition (Liu et al., 2014). 

The present study indicated that the expression level of the *EGFR, ERK-1,2* and *AKT-1,2* in glioma cell lines is medium and can induce cell growth in these cells. Additionally, increased expression of *miR-4731* significantly correlated with reduced expression of *EGFR, ERK-1,2 *and *AKT-1,2* at the mRNA levels and ERK-1,2 and AKT-1,2 at the protein levels. The difference in expression-eduction of *EGFR*, according to RT-qPCR in U-87 cell line is probably due to EGFR not mutated in this cell line (Patil et al., 2015). According to these results, We found that overexpression of *miR-4731* induced to increase the sub-G1 population in glioblastoma and leads to G1 arrest in cell cycle.

miR-4731 was previously reported to be suppressed in melanoma and following overexprression of miR-4731, multiple genes associated with cell cycle were regulates (Stark et al., 2016). In the present study, Through bioinformatics analysis, EGFR was predicted to contain a miR-4731 seed match at position 541-548 of the EGFR-3′UTR. To test whether miR-4731 directly targets 3′UTR of EGFR, we performed luciferase reporter assay. By measuring changes in the luciferase activity, we proved that miR-4731 directly targets 3′UTR of EGFR. In this study, we found new target for miR-4731 that may affect cell cycle. EGFR and downstream molecules such as AKT and ERK activate many biological outputs that are beneficial to cancer cell proliferation and progression through the cell cycle (Wee and Wang, 2017). Our study demonstrated that *miR-4731* overexpression could decrease the colony formation in glioma cells compared with the control groups. The present data indicated that miR-4731 attenuated cell proliferation in glioma cell lines.

In conclusion, the findings of the present study identified miR-4731 reduces *EGFR, AKT-1,2* and *ERK-1,2* expression in glioblastoma cell lines and induce cell cycle arrest and inhibit cell growth in these cells. Thus, *miR-4731* overexpression may be a potential therapeutic strategy in combination with conventtional therapy for the treatment of glioblastoma.
